# Ancient DNA of the Extinct Lava Shearwater (*Puffinus olsoni*) from the Canary Islands Reveals Incipient Differentiation within the *P. puffinus* Complex

**DOI:** 10.1371/journal.pone.0016072

**Published:** 2010-12-31

**Authors:** Oscar Ramirez, Juan Carlos Illera, Juan Carlos Rando, Jacob Gonzalez-Solis, Josep Antoni Alcover, Carles Lalueza-Fox

**Affiliations:** 1 Institute of Evolutionary Biology (CSIC-UPF), Barcelona, Spain; 2 Island Ecology and Evolution Research Group, IPNA-CSIC, La Laguna, Tenerife - Canary Islands (Spain); 3 Departamento de Biología Animal (UDI Zoología), Universidad de La Laguna, La Laguna, Tenerife - Canary Islands (Spain); 4 Departament de Biologia Animal, Universitat de Barcelona, Barcelona, Spain; 5 Institut Mediterrani d'Estudis Avançats (CSIC-UIB), Palma de Mallorca – Balearic Islands (Spain); Natural History Museum of Denmark, Denmark

## Abstract

**Background:**

The loss of species during the Holocene was, dramatically more important on islands than on continents. Seabirds from islands are very vulnerable to human-induced alterations such as habitat destruction, hunting and exotic predators. For example, in the genus *Puffinus* (family Procellariidae) the extinction of at least five species has been recorded during the Holocene, two of them coming from the Canary Islands.

**Methodology/Principal Findings:**

We used bones of the two extinct Canary shearwaters (*P. olsoni* and *P. holeae*) to obtain genetic data, for use in providing insights into the differentiation process within the genus *Puffinus*. Although mitochondrial DNA (mtDNA) cytochrome *b* sequences were successfully retrieved from four Holocene specimens of the extinct Lava shearwater (*P. olsoni*) from Fuerteventura (Canary Islands), the *P. holeae* specimens yielded no DNA. Only one haplotype was detected in *P. olsoni*, suggesting a low genetic diversity within this species.

**Conclusions:**

The phylogenetic analyses based on the DNA data reveal that: (i) the “*Puffinus puffinus* complex”, an assemblage of species defined using osteological characteristics (*P. puffinus*, *P. olsoni*, *P. mauretanicus*, *P. yelkouan* and probably *P. holeae*), shows unresolved phylogenetic relationships; (ii) despite the differences in body size and proportions, *P. olsoni* and the extant *P. puffinus* are sister species. Several hypotheses can be considered to explain the incipient differentiation between *P. olsoni* and *P. puffinus*.

## Introduction

In the recent history of the planet, humans have been a major underlying factor in determining extinction rates. In fact, the ongoing annihilation of vast numbers of species is known as the Holocene extinction [1]. In general, the ensuing loss of biodiversity is dramatically more pronounced on islands, than continents, as islands often have a higher number of endemic species per unit area, and specific adaptations of their biota that predispose them to extinction, including tameness, site faithfulness, flightlessness and reduced fecundity [2]–[9]. During the Holocene, more than 20 seabird extinctions and a higher number of local extirpations have been documented on islands around the world [7]–[10]. Phylogenetic relationships and causes of extinctions are often difficult to unravel, but recent studies using ancient DNA have greatly improved our understanding on the evolutionary history of these extinct species (e.g., [11]).

In most cases, decrease of distribution ranges or extinction has been related to human arrivals causing habitat destruction, hunting pressure and the introduction of exotic predators [2] [3] [5] [7] [10] [12]. Among seabirds, albatrosses and petrels (procellariforms) are particularly vulnerable to extinction due to their high breeding site fidelity, and lack of effective anti-predator behaviour (e.g., [13]). These species usually breed on islands free of predators. Thus, when predators are introduced, their limited behavioural plasticity becomes, in essence, an evolutionary trap, that can easily lead to extinction (e.g.,[14]). In fact, during the last 10,000 years, 56% of Holocene procellariform species have lost populations, and five *Puffinus* shearwater species with unclear evolutionary relationships have been reported to be extinguished. The reason for this is usually claimed to be the human arrival to the islands they inhabited [9] [10] [12] [15]. The genus *Puffinus* (family Procellariidae) is a diverse group of small and medium size birds (wings spanning 1.5–0.6 meters and weight of 170–700 grams) with a worldwide distribution [16]–[17]. Although in general recent phylogenetic studies of the group, based on mitochondrial gene trees of extant species, support previous morphological-based classifications [18]–[21], the phylogenetic relationships among some of the clades are still not well understood. For example, some of the monophyletic lineages such as *P. lherminieri*, *P. baroli* and *P. puffinus*, *P. yelkouan*, *P. mauretanicus* form unresolved polytomies within the genus *Puffinus* [21]. Such results could be explained by a recent diversification, lineage extinction and incomplete sampling of extant taxa [22] favoured by the remarkable philopatry exhibited by shearwater populations [23]–[25].

**Figure 1:**
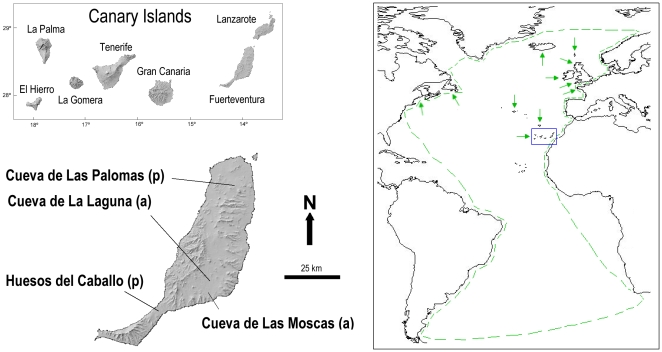
Geographic location of the Canary Islands (blue square), and breeding areas and distribution (green arrows and lines respectively) of *Puffinus puffinus*. The location of the sites where the bones for this work were collected is also showed. (a): archaeological site; (p): paleontological site.

The Dune (*P. holeae*) and Lava shearwater (*P. olsoni*) are two of the shearwater species that became extinct during the Holocene [26]–[27]. These are known to be former breeders in the Canary Islands, together with two other *Puffinus* species, the Manx shearwater (*P. puffinus*) (Figure 1) and the Little shearwater (*P. baroli*), which currently show a patchy distribution in the Canary Islands [28]. Distributions of *P. holeae* and *P. olsoni* were restricted to the Eastern Canary Islands (i.e., Lanzarote, Fuerteventura and islets around) [26]–[27] (Figure 1). According to the areas where bones were collected it is probable that they displayed different breeding behaviours. Bones of *P. olsoni* are abundant in caves located at recent lava fields [27], whereas remains of *P. holeae* are abundant at aeolianite formations or fossil dunes [26] [29].

It has been suggested that the extinction of *P. holeae* was directly linked to the aboriginal colonization of the Canary Islands, its last known record have been dated to 1,159±790 calibrated years (yr) before present (BP) [15]. *P. holeae* bones from sites located in the south of Fuerteventura are much older, dating to the Upper Pleistocene. Direct radiocarbon age on eggshells from one of these sites yielded an age of 32,100±1,100 14C yr BP [26].

In contrast, the known assemblage of *P. olsoni* remains is holocenic [27]. According to archaeological evidence, *P. olsoni* was also exploited as a food resource by the aboriginal Canarian people [30], but the extinction of this shearwater took place after 1270 AD, that is, more than one millennium after the arrival of the pre-Hispanic settlers. It has been suggested that the introduction of exotic mammals such as rats and cats after the European arrival to the Canary archipelago (14^th^ century) was the most probable cause of its extinction [9].

The morphological traits of the two extinct shearwaters have been thoroughly examined in relation to extant shearwaters. *P. olsoni* was intermediate in size (estimated weight range: 175–245 g; J.C.R., unpublished data), between *P. baroli* (170–225 g) and *P. puffinus* (375–459 g) [17]. *P. holeae* was larger than *P. olsoni*, with intermediate size (estimated weight range: 508–597 g; J.C.R., unpublished data) between *P. puffinus* and Cory's Shearwater, *Calonectris diomedea* (800–1,100 g) [17]. Irrespective to the differences in body sizes, some osteological traits (especially skull features) suggest that both extinct species were closely related to either *P. mauretanicus* or to *P. puffinus* [26]–[27]. However, their evolutionary relationships are still unclear and no attempt to reconstruct their phylogeny by means of molecular tools has been undertaken so far.

The aim of this study was to use ancient mtDNA sequences from bones of both Canary extinct species to: (i) to investigate their phylogenetic relationships within the shearwater group and estimate their divergence times; and (ii) to compare the phylogenetic information with the osteological characters in order to determine whether morphological differentiation is coincident with the genetic affinities obtained.

## Materials and Methods

### Samples

Fourteen bone fragments, from a minimum of five *P. olsoni* individuals, and 10 forelimb and hindlimb bone fragments (humerus, ulna, tibiotarsus and tarsometarsus), from a minimum of four *P. holeae* individuals, were used for DNA extraction. Materials were identified through direct comparison with bones of both species from the collections at the Zoology Department of La Laguna University (DZUL).

In order to increase the likelihood of recovering the maximum genetic variability of *P. olsoni*, three sites in Fuerteventura were selected for DNA analysis (Figure 1): three humeri plus one ulna, deriving from at least two specimens from Cueva de Las Palomas palaeontological site; one femur, one fragment of radius, one vertebra, four fragments of humerus plus one ulna, from at least two specimens from Cueva de Las Moscas archaeological site; and two humeri. from Cueva de La Laguna archaeological site (Figure 1). All samples were collected at the surface level. The recent aspect of the remains and the ^14^C ages of bones of this species from the two mentioned archaeological sites (1,290–1,440 and 750–969 calibrated yr respectively) [9] indicates a late Holocene age. No chronological information exists on bones from Cueva de Las Palomas, but based on the recent geological age of this volcano [31] the materials are estimated to be <10,000 years old. No material of putatively recent *P. holeae* are available for DNA analysis. Bones used for the extractions were collected at the site called Huesos del Caballo in the south of Fuerteventura. *P. holeae* eggshells collected from this paleontological site were previously dated to the Upper Pleistocene [26].

### Mitochondrial sequencing

*Puffinus* genomic DNA was isolated from bone powder in dedicated ancient DNA laboratories at the Institute of Evolutionary Biology (IBE) and at the University Pompeu Fabra (UPF) in Barcelona, by a proteinase-K extraction followed by a phenol-cloroform extractuib protocol and a Centricon-100 concentration column (Amicon), as described elsewhere [32]. No previous work with extant shearwaters had been conducted at these laboratories.

*Puffinus* specific primers were designed to amplify a fragment of 484 base pairs (bp) of the mitochondrial DNA (mtDNA) cytochrome *b* (cyt-b) gene. This was achieved through the amplification of five overlapping fragments of 173, 177, 119, 119 and 119 bp respectively, using a two-step PCR protocol [33]. Additionally, after unsuccessful amplifications, two shorter fragments of 75 and 102 bp were also tested in order to account for possible DNA degradation. Sequences of primers used for amplifying each one of the fragments targeted are reported in Table 1. Amplified products were purified with a gene clean silica method using the DNA Extration Kit (Fermentas, USA) and cloned using the Topo TA cloning kit (Invitrogen, The Netherlands). Insert-containing colonies were subjected to 30 cycles of PCR with M13 universal primers and subsequently sequenced with an Applied BioSystems 3100 DNA sequencer, at the Servei de Seqüenciació of the Universitat Pompeu Fabra (Barcelona).

**Table 1:** Primers used for amplification of a 484 bp fragment corresponding to the mtDNA cyt-b gene.

Primer Name	Sequence (5′-3′)	Product size
CytBPuf116F/CutBPuf253R	TTCGGCTCTCTCCTAGG/AAGAATGAGGCACCGTTTGC	173 bp
CytBPuf247F/CytBPuf382R	TGGTTGACTAATCCGAAACC/GGTAGGACATATCCTACGAAGGC	177 bp
CytBPuf368F/CytBPuf448R	AGGAGTCATCCTCCTACTCAC/TATGGGATGGCTGAGAATAG	119 bp
CytBPuf443F/CytBPuf522R	TGAGGAGCCACAGTCATCAC/GCGAAGAATCGGGTTAATGTG	119 bp
CytBPuf520F/CytBPuf601R	GGGATTCTCAGTAGACAACC/TTTGAGCCTGATTCATGGAG	119 bp
CytBPuf312F/CytBPuf345R	CACATCGGACGAGGATTCTAC/GAGTAGGAGGATGACTCCTGTG	75 bp
CytBPuf539F/CytBPuf601R	CCCACATTAACCCGATTCTT/TTTGAGCCTGATTCATGGAG	102 bp

### Phylogenetic Analyses

The ancient mtDNA cyt-b sequences obtained were compared to a dataset of 87 mtDNA cyt-b sequences originating from 34 extant species of the genus *Puffinus* gathered from NCBI GenBank (Table 2). Additionally, cyt-b sequences from *Calonectris diomedea*, *Lugensa brevirostris*, *Bulweria bulwerii*, *Diomedea epomophora*, *D. exulans*, *Oceanodroma furcata*, *O. leucorhoa*, *Pterodroma axillaris* and *Struthio camelus* were also obtained from GenBank to be used as outgroups (Table 2). Recent phylogenetic studied carried out with seabirds in the north Atlantic archipelagos [34]–[36] have estimated divergence times between lineages using the Kimura-2 correction, and suggest a mutation rate of 0.9% per million of years (mya) can be used for Procellariidae [37]. In order to compare our estimate with previous studies, genetic distances (corrected by the Kimura's two parameter evolution model) among taxa were obtained using MEGA 4.0 [38]. Then, divergence times were estimated using the aforementioned mutation rate of 0.9% per mya. Phylogenetic reconstruction was performed with Mr. Bayes 3.1.2. [39] [40]. The tree was rooted at the most phylogenetic distant outgroup species, *Struthio camelus*. The best model of nucleotide substitution was chosen by using the Bayesian Information Criteria model selection implemented in the program jModelTest version 0.1.1 [41]. Posterior distributions were obtained by four independent Monte Carlo Markov Chains (MCMCs), that included three heated chains and one cold chain of 10,000,000 iterations with the temperature set 0.2 each were run, and trees and model parameters were sampled every 1,000 generations. The convergence of the MCMCs was verified visually from the likelihood values but also we assessed the convergence with TRACER v. 1.5 [42]. The first quarter of sampled trees was discarded as burn-in, and the inference was drawn from the remaining trees. We repeated all MCMCs analyses twice in order to ensure the posterior probabilities were stable.

**Table 2:** Dataset of 87 mtDNA cyt-b sequences from 34 extant species of the genus *Puffinus* (extracted from the GenBank), plus the haplotype obtained from *P. olsoni* in this study (in bold), and the nine outgroup sequences.

Species	N	GenBank accession number	References
*P. assimilis*	1	AY219925	[21]
*P. atrodorsalis*	1	AY219965	[21]
*P. bailloni*	2	AY219963-AY219964	[21]
*P. baroli*	6	AF076080	[37]
		AY219934-AY219936	[21]
		AJ004206-AJ004207	[35]
*P. boydi*	1	AY219937	[21]
*P. bulleri*	1	AF076081	[37]
*P. carneipes*	1	AF076082	[37]
*P. colstoni*	4	AY219958-AY219959, AY219961-AY219962	[21]
*P. creatopus*	1	AF076083	[37]
*P. dichrous*	6	AY219949-AY219954	[21]
*P. elegans*	2	AY219932-AY219933	[21]
*P. gavia*	1	AY219977	[21]
*P. gravis*	1	U74354	[37]
*P. griseus*	1	U74353	[37]
*P. haurakiensis*	2	AY219930-AY219931	[21]
*P. huttoni*	2	AF076084	[37]
		AY219978	[21]
*P. kermadecensis*	3	AY219927-AY219929	[21]
*P. lherminieri*	8	AY219940-AY219945, AY219947-AY219948	[21]
*P. loyemilleri*	1	AY219946	[21]
*P. mauretanicus*	6	AJ004208-AJ004212	[35]
		AY219972	[21]
*P. myrtae*	2	AY219938-AY219939	[21]
*P. nativitatis*	2	AY219979	[21]
		AF076086	[37]
*P. newelli*	2	AY219974-AY219975	[21]
*P. nicolae*	3	AY219956-AY219957, AY219960	[21]
***P. olsoni***	**1**	**HQ651230**	**This study**
*P. opisthomelas*	2	AF076087	[37]
		AY219976	[21]
*P. pacificus*	1	AF076088	[37]
*P. persicus*	2	AY219966-AY219967	[21]
*P. polynesiae*	1	AY219955	[21]
*P. puffinus*	5	AJ004213-AJ004215	[37]
		AY219971	[21]
		U74355	[37]
*P. subalaris*	3	AY219968-AY219970	[21]
*P. temptator*	1	AY219980	[21]
*P. tenuirostris*	1	U74352	[37]
*P. tunneyi*	1	AY219926	[21]
*P. yelkouan*	10	AJ004216-AJ004224	[37]
		AY219973	[21]
*Bulweria bulwerii*	1	U74351	[37]
*Calonectris diomedea*	1	U74356	[37]
*Lugensa brevirostris*	1	U74357	[37]
*Pterodroma axillaris*	1	U74342	[37]
*Diomedea epomophora*	1	U48946	[49]
*Diomedea exulans*	1	U48947	[49]
*Oceanodroma furcata*	1	AF076063	[37]
*Oceanodroma leucorhoa*	1	AF076064	[37]
*Struthio camelus*	1	U76055	[50]

## Results and Discussion

The partial sequence (484 bp) of the cyt-*b* gene was obtained from four out of five specimens of *P.* olsoni (Figure S1). However, the *P. holae* samples yielded no successful amplifications (from the Huesos del Caballo site). This failure is not surprising, since these samples are the oldest tested, and the warm climatic conditions of the Canary Islands are highly unfavourable for long-term DNA preservation. Therefore, it can be expected *a priori* that many Holocene and pre-Holocene remains will have low or null endogenous DNA content. For instance, another Holocene specimen (*Myotragus balearicus*) from an equally unfavorable Mediterranean environment yielded only 0.27% endogenous DNA, as detected through unspecific shotgun sequencing [43]. Additionally, the remains of *P. holeae* come from a palaeontologic site exposed to climatic factors (rain, wind, sunlight), while those of *P. olsoni* come from caves, where the effect of these damaging agents is minimized.

**Figure 2:**
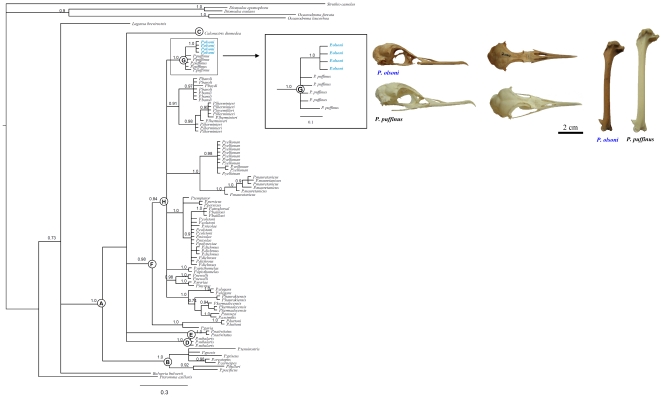
Tree topology obtained from Bayesian inferences. Numbers above nodes show the Bayesian posterior probability (>0.7). Letters show nodes discussed in the text. Cranium and humerus of *P. olsoni* (Holotype and Paratype; DZUL 2000 and 1903) and *P. puffinus* (DZUL 2756) are displayed to highlight the size differences between these sister taxa.

The DNA sequences from the four *P. olsoni* samples represent a unique and exclusive haplotype of the mtDNA cyt-*b* gene (Figure 2, Figure S1). Three of the four *P. olsoni*-specific substitutions correspond to either C to T or G to A transitions, which are commonly associated to DNA damage [44]. Nevertheless, we are confident of the truthfulness of these substitutions because: 1) they are reproducible among the four samples, 2) DNA from one *Puffinus* sample (*P. olsoni* 1) was independently extracted, amplified and sequenced in two dedicated ancient DNA laboratories for their authentication, and 3) about 50% of the fragments for each specimen have been replicated twice (Figure S1). Additional substitutions in one or few clones that are only present in one particular PCR but not in another PCR from the same sample can reasonably be attributed to DNA damage [44], and thus were not considered in the phylogenetic analyses.

jModelTest selected the Hasegawa Kishino Yano model (HKY +I+G). We confirmed with TRACER the concordance between runs obtained with the Bayesian inference. All parameters had effective sample size values above 240. The general topology obtained by performing Bayesian inference (Figure 2) supports previous phylogenetic assessment of the shearwaters, performed using different optimality criteria [18]–[21]. Our results do not seems to support the monophyly of the genus *Puffinus*, since *Calonectris diomedea* is grouped together to all *Puffinus* species with high nodal support (Figure 2, node A). The Bayesian Inference supports a monophyletic group of seven species (*P. tenuirostris*, *P. gravis*, *P. griseus*, *P. creatopus*, *P. carneipes*, *P. bulleri* and *P. pacificus*) that are a distinct and ancient lineage (node B). In contrast, *C. diomedea*, *P. subalaris* and *P. nativitatis* lineages show unresolved phylogenetic relationships to the rest of shearwaters species analyzed (nodes C, D, E and F). All *Puffinus* species included in node F are grouped together with high nodal support. The four *P. olsoni* individuals constitue a monophyletic clade with the five *P. puffinus* individuals, as supported by high Bayesian posterior probabilities (node G). However, the inclusion of *P. olsoni* in the phylogenetic analysis was unable to resolve the position of the *P. puffinus*-*P. olsoni* clade (node G) with respect to the large and monophyletic clade containing 27 *Puffinus* species (node H). This lack of resolution could be attributed to the rapid origin and radiation of this clade (node G) from the respective lineages within the monophyletic *Puffinus* group (node H).

Using the previously estimated mutation rate for Procellariidae of 0.9% per million of years [37], the time of the most recent common ancestor (MRCA) for *P. olsoni* and *P. puffinus* was estimated to be 600,000±400,000 years. Interestingly, the time for the split between *P. puffinus* and *P. olsoni* might be close to the diversification time estimated for the Cory's shearwater Palearctic clade (900,000–700,000 years ago) [34]. Recent phylogeographic studies have showed the influence of past climatic and geologic events on the patterns of genetic structure of many seabird species (e.g., [34] [35] [45]–[48]). Variation in marine productivity related to accessibility, availability and prey size could have produced specialization in foraging strategies and limited gene flow among seabird populations, thus favouring differentiation and speciation processes by allopatry and sympatry [21] [47].

According to some morphological traits, *P. olsoni* should be included within the so-called “*Puffinus puffinus* complex” (i.e. *P. puffinus*, *P. mauretanicus*, *P. yelkouan* and, probably, *P. holeae*). *Puffinus olsoni* is characterized by a lower and less bulky skull than their relatives. The premaxillary is very elongated with upper edges of the orbits being highly parallel and they display wide but flat humerus [27]. The mitochondrial DNA sequences suggest that the osteological affinities within this complex are not congruent with their phylogenetic relationships, due to the fact that these four taxa are not reciprocally monophyletic (Figure 2). Nevertheless, our results do indicate that, despite the conspicuous differences in size and proportions [27], *P. puffinus* and *P. olsoni* are sister species. Because both *Puffinus* species inhabited the Canary Islands (Figure 1), the recent split between *P. puffinus* and *P. olsoni* reveals an incipient differentiation process interrupted by the extinction of *P. olsoni*. Some authors [47] have suggested that the diversification process within the Madeiran storm-petrel (*Oceanodroma castro*), and perhaps in other seabird species, could be explained by allochrony (separation of populations by reproduction time). The timing of breeding within these seabirds varies among populations inhabiting the same archipelago. Albeit at present it is not possible to test this hypothesis with the extinct *P. olsoni*, a similar process could explain the recent split and genetic differentiation between this species and the sympatric *P. puffinus* in the Canary Islands. The differentiation process might have been favoured by the fact that both seabirds probably selected different habitats for nesting. *P. puffinus* selects laurel forest for nesting, but *P. olsoni* likely selected caves of lava fields in the semi-arid islands of the Canary archipelago [27]–[28]. The remarkable nesting philopatric behaviour of the *Puffinus* shearwaters (e.g., [23]–[25]) could have reinforced such differentiation.

The only cyt-*b* haplotype found in the four sequences obtained from *P. olsoni* suggests an unexpectedly low genetic diversity within this species, although incomplete sampling cannot be discarded. The fact that the sequences obtained originate from two different locations at the south of Fuerteventura (Figure 1), might provide support to the former hypothesis. However, it is difficult to to establish whether this low diversity is the result of incomplete sampling, of a recent bottleneck previous to its extinction, or of an older historical event. Further studies, that combine analysis of more individuals from more localities, radiocarbon dating on the bones in order to study possible temporal changes, and the sequencing of nuclear markers, will be needed to understand the evolutionary history of *P. olsoni*.

## Supporting Information

Figure S1 Alignment of a 484 bp fragment corresponding to the mtDNA cyt‐b gene obtained in the four samples of *P. olsoni*.
(DOC)
