# Supplementary material for: Ancient DNA of the Extinct Lava Shearwater (Puffinus olsoni) from the Canary Islands Reveals Incipient Differentiation within the P. puffinus Complex
Source: PLoS One. 2010 Dec 31;5(12):e16072. doi: 10.1371/journal.pone.0016072 (PMC3013140; doi:10.1371/journal.pone.0016072)
Supplement: Figure S1 — Alignment of a 484 bp fragment corresponding to the mtDNA cyt‐b gene obtained in the four samples of P. olsoni. (DOC) [file pone.0016072.s001.doc]

P.olsoni 1

U74355

ATGGCCCCAAACATCCGAAAATCCCATCCCCTACTAAAAATAGTCAACAACTCCTTAATCGACCTGCCTGCTCCCTCGAACATCTCTGCCTGATGAAACTTTGGCTCTCTCCTAGGCATCTGCCTACTAACCCAAATTTTAACTGGCCTACTACTAGCTATACACTACACTGCTGACACAACTCTAGCCTTTTCATCCGTCGCCCATACATGCCGAAACGTACAATATGGTTGACTAATCCGAAACCTACATGCAAACGGTGCCTCATTCTTCTTCATCTGTATTTACCTGCACATCGGACGAGGATTCTACTACGGCTCTTACCTATATAAAGAGACCTGAAACACAGGAGTCATCCTCCTACTCACTCTCATAGCAACTGCCTTCGTAGGATATGTCCTACC

AJ004215 TTCGGCTCTCTCCTAGGCATCTGCCTACTAACCCAAATTTTAACTGGCCTACTACTAGCTATACACTACACTGCTGATACAACTCTAGCCTTTTCATCCGTCGCCCATACATGCCGAAACGTACAATATGGTTGACTAATCCGAAACCTACATGCAAACGGTGCCTCATTCTT

1.1.1 CytBPuf116F ........................................................................................................................................ CytBPuf253R

1.1.2 ..................................T..................T......................C...........................................................

1.1.3 ........................................................................................................................................

1.1.4 ........................................................................................................................................

1.1.5 ........................................................................................................................................

1.1.6 ........................................................................................................................................

1.1.7 ........................................................................................................................................

1.1.8 ........................................................................................................................................

1.1.9 ........................................................................................................................................

1.1.10 .........................................................C..............T.C.............................................................

1.1.11 ........................................................................................................................................

1.1.12 .....................................................................................T..................................................

1.1.13 ........................................................................................................................................

1.1.14 .....................................................................................N............N....................

1.1.15 .................................................................N................N..N..................................................

1.1.16 ...........NNNN.........................................................................................................................

1.2.1 TTCGGCTCTCTCCTAGG............................................................c...........................................................................GCAAACGGTGCCTCATTCTT

CytBPuf116F CytBPuf253R

AJ004215 TGGTTGACTAATCCGAAACCTACATGCAAACGGTGCCTCATTCTTCTTCATCTGTATTTACCTGCACATCGGACGAGGATTCTACTACGGCTCTTACCTATATAAAGAGACCTGAAACACAGGAGTCATCCTCCTACTCACTCTCATAGCAACTGCCTTCGTAGGATATGTCCTACC

2.1.1 TGGTTGACTAATCCGAAACC................................N..........A.................................T........................................................GCCTTCGTAGGATATGTCCTACC

2.1.2 CytBPuf247 ...........................................A.................................N........................................................ CytBPuf382R

2.1.3 ...........................................A.................................T.........................................N..............

2.1.4 ...........................................A.................................T........................................................

2.1.5 ...........................................A.................................T........................................................

2.1.6 ...........................................A.................................T........................................................

2.1.7 ...........................................A.................................T........................................................

2.1.8 ........................................T..A.................................T.............T.......T..........T.......................

2.1.9 ........................................T..A.................................T........................................................

2.1.10 ...........................................A.................................T........................................................

2.1.11 ...........................................A.................................T........................................................

2.1.12 ...........................................A.................................T........................................................

2.1.13 ......................N..N.................A.....N...........N...............T....................N...................................

2.1.14 ...........................................A.........N.......................T...NN.NNN..N.N......N.............NN..........N..N....NN

2.2.1 TGGTTGACTAATCCGAAACC...........................................A.................................T........................................................GCCTTCGTAGGATATGTCCTACC

CytBPuf247 CytBPuf382R

AJ004215 AGGAGTCATCCTCCTACTCACTCTCATAGCAACTGCCTTCGTAGGATATGTCCTACC

3.1.1 AGGAGTCATCCTCCTACTCAC...................................T

3.1.2 CytBPuf368F .N.........N...N..N............N...T

3.2.1 AGGAGTCATCCTCCTACTCAC....................................

3.2.2 CytBPuf368F ...........AG.....A............G....

U74355 CTGAGGCCAAATATCATTCTGAGGAGCCACAGTCATCACCAACCTATTCTCAGCCATCCCATACATCGGCCAAACCCTCGTAGAATGAGCCTGAGGGGGATTCTCAGTAGACAACCCCACATTAACCCGATTCTTCGCCCTACATTTCCTCCTTCCTTTCATAATCGCAGGACTCACCCTAATTCACCTCACCTTCCTCCATGAATCAGGCTCAAACAATCCCCTTGGCATCGTATCAAACTGTGATAAAATCCCATTCCACCCCTATTTCACCCTAAAAGATATTCTAGGCTTCATACTCCTACTCCTTCCACTAACAACCCTAGCCCTATTTTCCCCAAACCTACTAGGAGATCCAGAAAATTTTACCCCAGCAAACCCGTTAGTCACACCTCCTC

CTGAGGCCAAATATCATTCTGAGGAGCCACAGTCATCACCAACCTATTCTCAGCCATCCCATA

3.1.1 ......................................TT...CTATTCTCAGCCATCCCATA

3.1.2 N.................N.......TT..........NT..T CytBPuf448R

3.2.1 ..............T............................CTATTCTCAGCCATCCCATA

3.2.2 ...........GG......C........T.............. CytBPuf448R

AJ004215 TGAGGAGCCACAGTCATCACCAACCTATTCTCAGCCATCCCATACATCGGCCAAACCCTCGTAGAATGAGCCTGAGGGGGATTCTCAGTAGACAACCCCACATTAACCCGATTCTTCGC

4.1.1 TGAGGAGCCACAGTCATCAC........................................................................T.....CACATTAACCCGATTCTTCGC

4.1.2 CytBPuf443F ..............................TT........................................T..... CytBPuf522R

4.1.3 ...............................T........................................T...T.

4.1.4 .............................A.............................A............T.....

4.1.5 ...........................................A.............A..............T.....

4.1.6 .........................................................AA.............T.....

4.1.7 ..G.....................................................................T.....

4.1.8 ........................................................................T.....

4.1.9 ........................................................................T.....

4.1.10 ........................................................................T.....

4.1.11 ........................................................................T.....

4.1.12 ........................................................................T.....

4.1.13 ........................................................................T.....

4.1.14 .............................A.............A............................T.....

4.1.15 .............N................N............N..N..N......................T.....

4.1.16 ........................................................................T.....

4.1.17 ........................................................................T.....

4.1.18 ...................N..........................................N.........T.....

4.1.19 ........................................................................T.....

4.1.20 ........................................................................T.....

4.1.21 ........................................................................T.....

4.1.22 ........................................................................T.....

4.1.23 ...............................................R.N.....N..............N.T.....

4.1.24 ...........................NN......................................A..A.T.....

4.1.25 .............................A.............A............................T.....

4.1.26 ...................................Y....................................T.....

4.1.27 .......................................................A................T.....

4.1.28 .........N.....T........................................................T.....

4.1.29 .....................................................N..................T.....

4.1.30 ........................................................................T.....

4.1.31 ...........T...............T.......................T.............T......T.....

4.1.32 ...........................................N............................T.....

4.1.33 ........................................................................T...N.

4.1.34 ........................................................................T....T

4.1.35 ........N..N............................................................T.....

4.1.36 ........................................N..A............................T.....

4.2.1 TGAGGAGCCACAGTCATCAC........................................................................T.....CACATTAACCCGATTCTTCGC

4.2.2 CytBPuf443F ..CTG...................................................................T..... CytBPuf522R

4.2.3 .....................................T..................................T.....

4.2.4 ........................................................................T.....

4.2.5 ...T...........................T........................................T.....

4.2.6 ...T.....T.....T..............T.........................................T.....

4.2.7 ...........................T............................................T.....

4.2.8 ...................T....................................................T.....

4.2.9 .....................................................A..................T.....

4.2.10 ........................................................................T.....

4.2.11 ........................................................................T.....

4.2.12 ...............................................A........................T.....

4.2.13 ........................................................................T.....

4.2.14 ...................TT...T..........................T..........................

AJ004215 GGGATTCTCAGTAGACAACCCCACATTAACCCGATTCTTCGCCCTACATTTCCTCCTTCCTTTCATAATCGCAGGACTCACCCTAATTCACCTCACCTTACTCCATGAATCAGGCTCAA

5.1.1 GGGATTCTCAGTAGACAACC.....CG..T..A.C.......T.....C...T..A.C......G..........C..T..T....GC........T.CCCTCCATGAATCAGGCTCAA

5.1.2 CytBPuf520F T.................................TT................................T..........C CytBPuf601R

5.1.3 T...............................................................................

5.2.1 ........................................................T......................C

5.2.2 GGGATTCTCAGTAGACAACC.................N.............................................................CCTCCATGAATCAGGCTCAA

5.2.3 CytBPuf520F ...............................................................................C CytBPuf601R

5.2.4 ...............................................................................C

P.olsoni 2 (vell)

U74355

ATGGCCCCAAACATCCGAAAATCCCATCCCCTACTAAAAATAGTCAACAACTCCTTAATCGACCTGCCTGCTCCCTCGAACATCTCTGCCTGATGAAACTTTGGCTCTCTCCTAGGCATCTGCCTACTAACCCAAATTTTAACTGGCCTACTACTAGCTATACACTACACTGCTGACACAACTCTAGCCTTTTCATCCGTCGCCCATACATGCCGAAACGTACAATATGGTTGACTAATCCGAAACCTACATGCAAACGGTGCCTCATTCTTCTTCATCTGTATTTACCTGCACATCGGACGAGGATTCTACTACGGCTCTTACCTATATAAAGAGACCTGAAACACAGGAGTCATCCTCCTACTCACTCTCATAGCAACTGCCTTCGTAGGATATGTCCTACC

AJ004215 TTCGGCTCTCTCCTAGGCATCTGCCTACTAACCCAAATTTTAACTGGCCTACTACTAGCTATACACTACACTGCTGATACAACTCTAGCCTTTTCATCCGTCGCCCATACATGCCGAAACGTACAATATGGTTGACTAATCCGAAACCTACATGCAAACGGTGCCTCATTCTT

1.1.1 TTCGGCTCTCTCCTAGG........................................................................................................................................GCAAACGGTGCCTCATTCTT

CytBPuf116F CytBPuf253R

AJ004215 TGGTTGACTAATCCGAAACCTACATGCAAACGGTGCCTCATTCTTCTTCATCTGTATTTACCTGCACATCGGACGAGGATTCTACTACGGCTCTTACCTATATAAAGAGACCTGAAACACAGGAGTCATCCTCCTACTCACTCTCATAGCAACTGCCTTCGTAGGATATGTCCTACC

2.1.1 TGGTTGACTAATCCGAAACC................................N..........A.................................T........................................................GCCTTCGTAGGATATGTCCTACC

CytBPuf247F CytBPuf382R

AJ004215 AGGAGTCATCCTCCTACTCACTCTCATAGCAACTGCCTTCGTAGGATATGTCCTACC

3.1.1 AGGAGTCATCCTCCTACTCAC..............................TT..TT

3.1.2 CytBPuf368F .................NNN..........TT..TT

U74355 CTGAGGCCAAATATCATTCTGAGGAGCCACAGTCATCACCAACCTATTCTCAGCCATCCCATACATCGGCCAAACCCTCGTAGAATGAGCCTGAGGGGGATTCTCAGTAGACAACCCCACATTAACCCGATTCTTCGCCCTACATTTCCTCCTTCCTTTCATAATCGCAGGACTCACCCTAATTCACCTCACCTTCCTCCATGAATCAGGCTCAAACAATCCCCTTGGCATCGTATCAAACTGTGATAAAATCCCATTCCACCCCTATTTCACCCTAAAAGATATTCTAGGCTTCATACTCCTACTCCTTCCACTAACAACCCTAGCCCTATTTTCCCCAAACCTACTAGGAGATCCAGAAAATTTTACCCCAGCAAACCCGTTAGTCACACCTCCTC

CTGAGGCCAAATATCATTCTGAGGAGCCACAGTCATCACCAACCTATTCTCAGCCATCCCAT

3.1.1 T..........................................CTATTCTCAGCCATCCCAT

3.1.2 T.......................................... CytBPuf448R

AJ004215 TGAGGAGCCACAGTCATCACCAACCTATTCTCAGCCATCCCATACATCGGCCAAACCCTCGTAGAATGAGCCTGAGGGGGATTCTCAGTAGACAACCCCACATTAACCCGATTCTTCGC

4.1.1 TGAGGAGCCACAGTCATCAC..................NNN-..T..T.......TTT.T................................T....TCACATTAACCCGATTCTTCGC

4.1.2 CytBPuf443F ................N.T.....T..........TT...................................T..... CytBPuf522R

4.1.3 .....................................T..................................T..TT.

4.1.4 ..............TT..T.....................................................T....T

4.1.5 ...........................T.......TTT.T................................T....T

4.1.6 ...........................T.......TTT.T................................T.....

4.1.7 ..............T......................T..................................T.....

4.1.8 .....................................T..................................T.....

4.1.9 ..................T.....T.....T....TT.....................NNNN.NN.......T.....

4.1.10 .....................................T...............NNNNNNNNNN-G.......T....T

4.1.11 ..................T...........T....TT.........................................

4.1.12 ...........................T.......TTT.T................................T....T

4.1.13 ..............T......................T..................................T.....

4.1.14 ...........................T.......TTT.T................................T.....

4.1.15 ........................................................................T.....

4.1.16 ...........................T.......TTT.T................................T....T

4.1.17 ...........................T.......TTT.T................................T....T

4.1.18 .........N.................T.......TTT.T................................T....T

4.1.19 ...........................T.......TTT.T................................T....T

4.1.20 .....................................T..................................T.....

4.1.21 ........................................................................T.....

4.1.22 ...........................T.......TTT.T................................T.....

4.1.23 ..............T......................T..................................T.....

4.1.24 ..................T...........T....TTC..................................T.....

4.1.25 ...........................T.......TTT.T................................T....T

4.1.26 .....................................T..................................T.....

4.1.27 .....................................T..................................T..T..

4.1.28 ........................................................................T.....

4.1.29 ........................................................................T.....

AJ004215 GGGATTCTCAGTAGACAACCCCACATTAACCCGATTCTTCGCCCTACATTTCCTCCTTCCTTTCATAATCGCAGGACTCACCCTAATTCACCTCACCTTACTCCATGAATCAGGCTCAA

5.1.1 GGGATTCTCAGTAGACAACCT...........................................................TTG................CCTCCATGAATCAGGCTCAA

CytBPuf520F CytBPuf601R

5.2.1 GGGATTCTCAGTAGACAACCT...........................................................TT.................CCTCCATGAATCAGGCTCAA

CytBPuf520F CytBPuf601R

P.olsoni 3 (Mosca)

U74355

ATGGCCCCAAACATCCGAAAATCCCATCCCCTACTAAAAATAGTCAACAACTCCTTAATCGACCTGCCTGCTCCCTCGAACATCTCTGCCTGATGAAACTTTGGCTCTCTCCTAGGCATCTGCCTACTAACCCAAATTTTAACTGGCCTACTACTAGCTATACACTACACTGCTGACACAACTCTAGCCTTTTCATCCGTCGCCCATACATGCCGAAACGTACAATATGGTTGACTAATCCGAAACCTACATGCAAACGGTGCCTCATTCTTCTTCATCTGTATTTACCTGCACATCGGACGAGGATTCTACTACGGCTCTTACCTATATAAAGAGACCTGAAACACAGGAGTCATCCTCCTACTCACTCTCATAGCAACTGCCTTCGTAGGATATGTCCTACC

AJ004215 TTCGGCTCTCTCCTAGGCATCTGCCTACTAACCCAAATTTTAACTGGCCTACTACTAGCTATACACTACACTGCTGATACAACTCTAGCCTTTTCATCCGTCGCCCATACATGCCGAAACGTACAATATGGTTGACTAATCCGAAACCTACATGCAAACGGTGCCTCATTCTT

1.1.1 TTCGGCTCTCTCCTAGG......................................................................................................T.................................GCAAACGGTGCCTCATTCTT

1.1.2 CytBPuf116F ......................................................................................................T................................. CytBPuf253R

1.1.3 ......................................................................................................T.................................

1.1.4 ......................................................................................................T.................................

1.1.5 ........................................................................................................................................

1.1.6 ........................................................................................................................................

1.1.7 ........................................................................................................................................

1.1.8 ........................................................................................................................................

1.1.9 .................N.....................................................T..............................T.................................

1.1.10 ............................................................................................T.........T.................................

AJ004215 TGGTTGACTAATCCGAAACCTACATGCAAACGGTGCCTCATTCTTCTTCATCTGTATTTACCTGCACATCGGACGAGGATTCTACTACGGCTCTTACCTATATAAAGAGACCTGAAACACAGGAGTCATCCTCCTACTCACTCTCATAGCAACTGCCTTCGTAGGATATGTCCTACC

2.1.1 TGGTTGACTAATCCGAAACC...........................................A.................................T........................................................GCCTTCGTAGGATATGTCCTACC

2.1.2 CytBPuf247F .........................N.................A.................................T..................N..................................... CytBPuf382R

2.1.3 ...........................................A.................................T........................................................

2.1.4 ...........................................A.................................T........................................................

2.1.5 ...........................................A.................................T........................................................

2.1.6 ...........................................A.................................T......................................N.................

2.1.7 ................................N..........A................................NNN..................................N..N................N

2.1.8 ...........................................A.................................T.......................................

2.1.9 ...........................................A.................................T..................................

2.1.10 ...........................................A.................................T................................

AJ004215 AGGAGTCATCCTCCTACTCACTCTCATAGCAACTGCCTTCGTAGGATATGTCCTACC

3.1.1 AGGAGTCATCCTCCTACTCAC....................................

3.1.2 CytBPuf368F ....................................

3.1.3 ....................................

3.1.4 ...................N..N.............

3.1.5 .................N.N................

3.1.6 ....................................

3.1.7 ....................................

3.1.8 ....................................

3.1.9 ....................................

3.1.10 .............N......................

3.1.11 ....................................

3.1.12 ....................................

3.1.13 ....................................

3.1.14 ....................................

3.1.15 ...................................T

3.1.16 ........T...........................

3.1.17 ....................................

3.1.18 .............N......................

U74355 CTGAGGCCAAATATCATTCTGAGGAGCCACAGTCATCACCAACCTATTCTCAGCCATCCCATACATCGGCCAAACCCTCGTAGAATGAGCCTGAGGGGGATTCTCAGTAGACAACCCCACATTAACCCGATTCTTCGCCCTACATTTCCTCCTTCCTTTCATAATCGCAGGACTCACCCTAATTCACCTCACCTTCCTCCATGAATCAGGCTCAAACAATCCCCTTGGCATCGTATCAAACTGTGATAAAATCCCATTCCACCCCTATTTCACCCTAAAAGATATTCTAGGCTTCATACTCCTACTCCTTCCACTAACAACCCTAGCCCTATTTTCCCCAAACCTACTAGGAGATCCAGAAAATTTTACCCCAGCAAACCCGTTAGTCACACCTCCTC

CTGAGGCCAAATATCATTCTGAGGAGCCACAGTCATCACCAACCTATTCTCAGCCATCCCAT

3.1.1 ...........................................CTATTCTCAGCCATCCCAT

3.1.2 ........................................... CytB448R

3.1.3 .......N...................................

3.1.4 ...........................................

3.1.5 ...........................................

3.1.6 ...........................................

3.1.7 ...........................................

3.1.8 ...........................................

3.1.9 ..................N....N.N.................

3.1.10 ...........................................

3.1.11 ..................N........................

3.1.12 ...........................................

3.1.13 ...........................................

3.1.14 ..................N....N...................

3.1.15 ...........................................

3.1.16 ...........................................

3.1.17 ........................N..................

3.1.18 .................N...NNNNN.................

AJ004215 TGAGGAGCCACAGTCATCACCAACCTATTCTCAGCCATCCCATACATCGGCCAAACCCTCGTAGAATGAGCCTGAGGGGGATTCTCAGTAGACAACCCCACATTAACCCGATTCTTCGC

4.1.1 TGAGGAGCCACAGTCATCAC.................................................................T......T.....CACATTAACCCGATTCTTCGC

4.1.2 CytBPuf443F ........................................................................T..... CytBPuf522R

4.1.3 .......................................................AA...............T.....

4.1.4 ........................................................................T.....

4.1.5 ........................................................................T.....

4.1.6 .........N..............................................................T.....

4.1.7 .........N.................................................NN...........T.....

4.1.8 ...........................................................NA...........T.....

4.1.9 .........N.........................................N....................T.....

4.1.10 .............................................G.........N................T.....

4.1.11 ...........................................................ANN.N-.......T.....

4.2.1 TGAGGAGCCACAGTCATCAC..............................T.........................................T.....CACATTAACCCGATTCTTCGC

4.2.2 CytBPuf443F ........................................................................T..... CytBPuf522R

4.2.3 ........................................................................T.....

4.2.4 ........................................................................T.....

4.2.5 ........................................................................T.....

4.2.6 ........................................................................T.....

4.2.7 ........................................................................T.....

4.2.8 ........................................................................T.....

4.2.9 ...N................N...................................................T.....

4.2.10 ....................................T...................................T.....

4.2.11 ..................................................................N.....T.....

4.2.12 ........................................................................T....T

4.2.13 ........................................................................T.....

4.2.14 ........................................................................T.....

4.2.15 ........................................................................T.....

4.2.16 ........................................................................T.....

AJ004215 GGGATTCTCAGTAGACAACCCCACATTAACCCGATTCTTCGCCCTACATTTCCTCCTTCCTTTCATAATCGCAGGACTCACCCTAATTCACCTCACCTTACTCCATGAATCAGGCTCAA

5.1.1 GGGATTCTCAGTAGACAACC.......................................................G.......................TCTCCATGAATCAGGCTCAA

CytBPuf520F CytBPuf601R

5.2.1 GGGATTCTCAGTAGACAACC....................NN.........................................................CCTCCATGAATCAGGCTCAA

5.2.2 CytBPuf520F ................N...................................NNNNNNNNNNN................C CytBPuf601R

5.2.3 ...........................................................................T...T

5.2.4 ..........................................................N....................C

5.2.5 ................T......T...............T................T....TT................T

5.2.6 ...............................................................................C

5.2.7 ........................................................NN.N..N................C

5.2.8 .T.......T.....................................................................T

5.2.9 ......................................................N.N......................C

5.2.10 .T.......T...........................................NNNN.NNNNNN...............T

P.olsoni 4 (Mujer)

U74355

ATGGCCCCAAACATCCGAAAATCCCATCCCCTACTAAAAATAGTCAACAACTCCTTAATCGACCTGCCTGCTCCCTCGAACATCTCTGCCTGATGAAACTTTGGCTCTCTCCTAGGCATCTGCCTACTAACCCAAATTTTAACTGGCCTACTACTAGCTATACACTACACTGCTGACACAACTCTAGCCTTTTCATCCGTCGCCCATACATGCCGAAACGTACAATATGGTTGACTAATCCGAAACCTACATGCAAACGGTGCCTCATTCTTCTTCATCTGTATTTACCTGCACATCGGACGAGGATTCTACTACGGCTCTTACCTATATAAAGAGACCTGAAACACAGGAGTCATCCTCCTACTCACTCTCATAGCAACTGCCTTCGTAGGATATGTCCTACC

AJ004215 TTCGGCTCTCTCCTAGGCATCTGCCTACTAACCCAAATTTTAACTGGCCTACTACTAGCTATACACTACACTGCTGATACAACTCTAGCCTTTTCATCCGTCGCCCATACATGCCGAAACGTACAATATGGTTGACTAATCCGAAACCTACATGCAAACGGTGCCTCATTCTT

1.1 TTCGGCTCTCTCCTAGG.................N......................................................................................................................GCAAACGGTGCCTCATTCTT

1.2 CytBPuf116F ........................................................................................................................................ CytBPuf253R

1.3 ........................................................................................................................................

1.4 ........................................................................................................................................

1.5 ........................................................T...............................................................................

1.6 ........................................................................................................................................

1.7 ........................................................................................................................................

1.8 ........................................................................................................................................

1.9 ........................................................................................................................................

1.10 ........................................................................................................................................

1.11 .................N........NN............................................................................................................

1.12 .............................................................................................G..........................................

AJ004215 TGGTTGACTAATCCGAAACCTACATGCAAACGGTGCCTCATTCTTCTTCATCTGTATTTACCTGCACATCGGACGAGGATTCTACTACGGCTCTTACCTATATAAAGAGACCTGAAACACAGGAGTCATCCTCCTACTCACTCTCATAGCAACTGCCTTCGTAGGATATGTCCTACC

2.1.1 TGGTTGACTAATCCGAAACC...........................................A.................................T........................................................GCCTTCGTAGGATATGTCCTACC

2.1.2 CytBPuf247F ...........................................A.................................T........................................................ CytBPuf382R

2.1.3 ...........................................A.................................T........................................................

2.1.4 ...........................................A.................................T........................................................

2.1.5 ...........................................A.................................T........................................................

2.1.6 ...........................................A.................................T........................................................

2.1.7 ...........................................A.................................T........................................................

2.1.8 ...........................................A.................................T........................................................

2.1.9 ...........................................A.................................T......................N...............N.................

2.1.10 ...........................................A.................................T................................

AJ004215 AGGAGTCATCCTCCTACTCACTCTCATAGCAACTGCCTTCGTAGGATATGTCCTACC

3.1.1 AGGAGTCATCCTCCTACTCAC...................N................

3.1.2 CytBPuf368F ...................N................

3.1.3 ................NNNN.....N..........

3.1.4 ...................N..N.............

3.1.5 ....................................

3.1.6 ....................................

3.1.7 ....................................

3.1.8 ....................................

U74355 CTGAGGCCAAATATCATTCTGAGGAGCCACAGTCATCACCAACCTATTCTCAGCCATCCCATACATCGGCCAAACCCTCGTAGAATGAGCCTGAGGGGGATTCTCAGTAGACAACCCCACATTAACCCGATTCTTCGCCCTACATTTCCTCCTTCCTTTCATAATCGCAGGACTCACCCTAATTCACCTCACCTTCCTCCATGAATCAGGCTCAAACAATCCCCTTGGCATCGTATCAAACTGTGATAAAATCCCATTCCACCCCTATTTCACCCTAAAAGATATTCTAGGCTTCATACTCCTACTCCTTCCACTAACAACCCTAGCCCTATTTTCCCCAAACCTACTAGGAGATCCAGAAAATTTTACCCCAGCAAACCCGTTAGTCACACCTCCTC

CTGAGGCCAAATATCATTCTGAGGAGCCACAGTCATCACCAACCTATTCTCAGCCATCCCATA

3.1.1 .........................................N.CTATTCTCAGCCATCCCATA

3.1.2 ........................................... CytBPuf448R

3.1.3 ...........................................

3.1.4 ...........................................

3.1.5 ...........................................

3.1.6 ...........................................

3.1.7 ...........................................

3.1.8 ...........................................

AJ004215 TGAGGAGCCACAGTCATCACCAACCTATTCTCAGCCATCCCATACATCGGCCAAACCCTCGTAGAATGAGCCTGAGGGGGATTCTCAGTAGACAACCCCACATTAACCCGATTCTTCGC

4.1.1 TGAGGAGCCACAGTCATCAC........................................................................T.....CACATTAACCCGATTCTTCGC

4.1.2 CytBPuf443F .........N..............................................................T..... CytBPuf522R

4.1.3 ........................................................................T.....

4.1.4 ........................................................................T.....

4.1.5 ........................................................................T.....

4.1.6 .........N..............................................................T.....

4.1.7 ........................................................................T.....

4.1.8 .........N...............................................NNNN...........T.....

4.1.9 .......C................................................................T.....

4.2.1 TGAGGAGCCACAGTCATCAC.N............................................N..N......................T.....CACATTAACCCGATTCTTCGC

4.2.2 CytBPuf443F ........................................................................T..... CytBPuf522R

4.2.3 ........................................................................T.....

4.2.4 ........................................N..........................N....T.....

4.2.5 ........................................................................T.....

4.2.6 ........................................................................T.....

4.2.7 ........................................................................T.....

4.2.8 ........................................................................T.....

4.2.9 ........................................................................T.....

4.2.10 ........................................................................T.....

4.2.11 ........................................................................T.....

4.2.12 ........................................................................T.....

4.2.13 ........................................................................T.....

4.2.14 ........................................................................T.....

4.2.15 .........N.N............................................................T.....

AJ004215 GGGATTCTCAGTAGACAACCCCACATTAACCCGATTCTTCGCCCTACATTTCCTCCTTCCTTTCATAATCGCAGGACTCACCCTAATTCACCTCACCTTACTCCATGAATCAGGCTCAA

5.1.1 GGGATTCTCAGTAGACAACC........................................N......................................CCTCCATGAATCAGGCTCAA

5.1.2 CytBPuf520F ...............................................................................C CytBPuf601R

5.1.3 ...............................................................................C

5.2.1 GGGATTCTCAGTAGACAACC...............................................................................CCTCCATGAATCAGGCTCAA

5.2.2 CytBPuf520F ...............................................................................C CytBPuf601R

5.2.3 ...............................................................................C

5.2.4 ...............................................................................C

5.2.5 ..............................................................N................C

5.2.6 ...............................................................................C

5.2.7 ...............................................................................C

5.2.8 ...............................................................................C

5.2.9 ...............................................................................C

5.2.10 ..............................................................N................C

5.2.11 ...............................................................................C

5.2.12 ........................................................N......................C

5.2.13 ...............................................................................C

5.2.14 ........................................................NNNN...................C

5.3.1 GGGATTCTCAGTAGACAACC....................N..........................................................CCTCCATGAATCAGGCTCAA

5.3.2 CytBPuf520F .......................N......N.............................N..N......N........C CytBPuf601R
